# Fruits and Berries

**Published:** 1870-07

**Authors:** 


### FRUITS AND BERRIES

May be taken all summer with the utmost freedom at breakfast and dinner. To derive the fullest benefit from them they should be ripe, raw, perfect and fresh, and eaten in that state without cream, or milk, or sugar; but in their raw, natural condition without taking any fluid whatever within an hour or two of eating them, because fluids dilute their acidity and thus weaken its effect on the system, when it is for their acid qualities that they are most valuable; besides, if fluids are taken largely, especially milk, they are liable to cause fermentation in the stomach, ending in cholera or cholera morbus. General Taylor lost his life in that way. The present executive had lately a serious attack of illness from this same cause; especially are berries and cream hazardous in the latter part of the day. If taken as above suggested, in their natural, raw, ripe state, it is difficult to take too many in summer, for they are essentially anti-bilious, and cooling and nutritious.

